# Fast UV-Curable Zwitter-Wettable Coatings with Reliable Antifogging/Frost-Resisting Performances

**DOI:** 10.3390/biomimetics7040162

**Published:** 2022-10-13

**Authors:** Hao Zhong, Xiaoxiao Liu, Boxin Yu, Shengzhu Zhou

**Affiliations:** 1 Agriculture College, Yanbian University, Yanbian 133002, China; 2 Institute of Animal Husbandry and Veterinary Medicine, Jilin Academy of Agricultural Sciences, Changchun 130119, China; 3 Department of General Practice, The First Hospital of Jilin University, Changchun 130021, China; 4 Department of Anesthesiology, The Second Hospital of Jilin University, Changchun 130061, China

**Keywords:** antifogging, frost-resisting, zwitter-wettable, UV illumination, acrylate coating

## Abstract

Antifogging surfaces with unique properties to migrate severe fog formation have gained extensive interest, which is of particular interest for transparent substrates to obtain high visibility and transparency. To date, a large number of strategies including superhydrophilic or superhydrophobic surfaces and titanium dioxide (TiO_2_)-based composite coatings have been developed based on different mechanisms. Although these surfaces exhibit effective antifogging properties, the rigid nanostructures, cumbersome preparation, and the need for UV light excitation largely limit their widespread applications. Herein, we report a zwitter-wettable antifogging and frost-resisting coating through a fast UV-curable cross-linking of copolymer with benzophenone groups. A series of random copolymers consisting of hydrophilic hydroxyethyl methacrylate (HEA), hydrophobic methyl methacrylate (MMA), and benzophenone-based acrylate units are developed by thermally triggered free-radical polymerization. Upon UV light irradiation, a highly efficient antifogging/frost-resisting coating is covalently bonded on a polycarbonate plate surface, maintaining a light transmission higher than 85%, which was confirmed in both high and low temperature anti-fog tests. Moreover, the wetting behaviors reveal that the antifogging performance exhibited by the zwitter-wettable surface mainly relies on its surface water-adsorbing capability to imbibe condensed water vapor on the surface outmost layer. Notably, the antifogging/frost-resisting behaviors can be well regulated by adjusting the hydrophilic/hydrophobic units, due to the proper balance between the water-adsorption and coating stability. Owing to its simplicity, low-cost preparation and high efficiency, this UV-curable acrylate antifogging coating may find a wide range of applications in various display devices in analytical and detection instruments.

## 1. Introduction

Fogging and frosting are prevalent in nature and cause a lot of inconvenience to humans daily [1,2,3,4,5,6,7,8,9,10]. Owing to the rapid changes in temperature and humidity, saturated water vapor in the air condenses and forms fog droplets on the solid substrates [11]. The formation of fog droplets will not only cause surface wetting, but also has a great impact on the light transmission of the transparent materials, resulting in a significant reduction in their view clarity [12,13]. For example, the presence of a fog layer can largely reduce the solar energy conversion rate of solar panels [14,15,16]. Severe fog formation can blur the vision of vehicle drivers, which can easily cause traffic accidents [17]. In addition, in the field of medical testing, fogging of the testing lens during surgery can even cause catastrophic medical accidents [9,18]. So far, a large number of antifogging strategies have been designed and prepared to mitigate severe fogging and frosting. Among those, superhydrophilic surfaces that can get extremely low water contact angles within 0.5 s or less, have the ability to significantly reduce light scattering by allowing water to spread into a thin film [19,20]. For conventional superhydrophilic surfaces, both high surface energy and suitable roughness scales are extremely necessary to enhance the surface super-wetting behaviors [21]. Till now, various techniques such as plasma etching [22], layer-by-Layer (LbL) [23], templating method [24] have been adopted to develop the superhydrophilic antifogging surfaces. However, those techniques generally require either complicated procedures or special instruments to get the appropriate surface roughness. Additionally, the superhydrophilic surfaces can also be prepared by introducing the photochemically active materials (e.g., TiO_2_) into its coating [25,26]. However, most of those coatings must be exposed under UV illumination to obtain the superhydrophilic anti-fogging properties. On the other hand, some superhydrophobic surfaces also displayed antifogging behaviors due to its super-repellency against water droplets [27,28,29]. Considering the micro-scale fog droplets formed on the surface, only some special superhydrophobic surfaces with precisely controlled roughness and topography can exhibit the qualified antifogging ability. Typical superhydrophobic surfaces are opaque and have very low light transmission, not to mention that the micro and nano structures are easily damaged and not easy to handle, all of which lead to difficulties in the application of the anti-fogging properties of superhydrophobic surfaces.

Recently, some coatings with zwitter-wettability have been reported to have good anti-fogging behavior [30,31,32,33,34,35]. Unlike the previously reported superhydrophilic or superhydrophobic surfaces, these zwitter-wettable coatings have moderate water absorption capacity. The anti-fogging mechanism of the coatings is believed to be that their coatings can strongly adsorb water vapor to their bulk materials rather than forming condensed water droplets on the surfaces, finally resulting in a totally clear coating surface. Some zwitterionic wettable coatings were prepared by grafting with oligomers consisting of perfluoroalkyl and polyethylene glycol (PEG) segments [36] or by a layer-by-layer (LbL) assembly method based on chitosan/Nafion systems [37]. More recently, some polymeric coatings with a semi-interpenetrating polymer network (SIPN) have been developed, through a binary or terpolymer acrylic polymers. Moreover, some zwitter-wettable coatings with both antifogging/antibacterial performances can also be developed by the combination of a cationic copolymer and a hydrophilic copolymer [38,39]. Although good anti-fog properties have been obtained for these coatings, there is still a strong demand for preparing these wettable coatings by a more rapid and convenient method.

Although the coatings exhibit effective antifogging performances because of the water-adsorbing behaviors, the inhaled water may turn into ice crystal under an extremely cold condition below the water freezing point, which inevitably decrease the light transmission. Nature always offers immense inspirations for creating functional material and surfaces. The roots of some overwintering plants, with high water content, can tolerate extremely cold temperatures. The key to this property is that the water present in the roots is in a nonfreezing or intermediate water states, thus avoiding the formation of ice crystal as well as the damage of plant cells [40,41,42].

Inspired by this concept, we herein develop a zwitter-wettable acrylate coating with antifogging and frost-resisting performances through a facile UV-curable cross-linking of copolymer with benzophenone groups. Initially, a series of copolymers consisting of hydrophilic hydroxyethyl methacrylate (HEA), hydrophobic methyl methacrylate (MMA), and benzophenone-based acrylate (BP-Acrylate) units were synthesized by thermally triggered radical polymerization. The hydrophilic-hydrophobic balance of the copolymers can be well adjusted by adjusting the molar ratio of HEA/MMA. The structure and molecular weight of the prepared copolymers were investigated by nuclear magnetic resonance (NMR) and gel permeation chromatography (GPC). Subsequently, the copolymers were coated on the surface of PC plates to obtain covalently bonded anti-fogging coatings under UV light. The anti-fog and anti-frost properties were investigated, and the antifogging mechanism was studied by surface wetting behavior.

## 2. Materials and Methods

### 2.1. Materials

Hydroxyethyl methacrylate (HEA, 98%) and hydrophobic methyl methacrylate (99%) were obtained from Aladdin (Shanghai, China). Control PC plates (BN-HFR, 2 mm in thickness) were provided by Bornsun (Shenzhen Bornsun Industrial Co, Shenzhen, China). 4-Benzoylphenyl acrylate (4-BP acrylate, 98%) was bought from Anhui Yousheng Biotechnology Company. 2,2′-azobis(2-methylpropionitrile) (AIBN, 98%) were purchased from Sigma-Aldrich (Shanghai, China). All solvents including N,N-dimethylformamide (DMF, 98%), acetone (99%) Tetrahydrofuran (THF, 98%) were purchased from Aladdin (Shanghai, China). All other chemicals were analytical grade reagents and used without any further purification. Deionized water (18.25 MΩ cm) was made in the laboratory.

### 2.2. Synthesis of BP-Acrylate-Based Copolymers

A series of acrylate copolymers were synthesized via free radical copolymerization containing HEA, MMA, and 4-BP acrylate monomers, with AIBN as the thermal initiator. Three acrylate monomers of HEA (3.0 g), MMA (7.0 g) and 4-BP acrylate (0.5 g) and AIBN (0.21g, ~2% of total mass weight of monomers) were dissolved in 10 mL DMF to get a homogeneous solution. The mixture was maintained at 60 °C for 12 h, and then dialyzed against distilled water using 5 kDa MWCO dialysis tubing with a regenerated cellulose membrane for 24 h with 3 water changes to remove the organic solvent and unreacted monomer. The sample was labeled as P-30% according to the mass percentage of HEA (30 wt.%) to the total mass weight of HEA and MMA. Similarly, other products with different mass percentage of HEA (50, 70, and 90 wt.%) to the total mass weight of HEA and MMA were also prepared and labeled as P-50%, P-70% P-90%, accordingly. All the purified copolymers (P-30%, P-50%, P-70% P-90%) were dissolved in D6-DMSO to get a uniform solution, respectively. ^1^H-NMR measurements were carried out on 400 MHz NMR spectrometer from Bruker Biospin. About 100 mg of polymer was dissolved in 1 mL in *d*6-DMSO solvent. The related chemical structures were characterized by the ^1^H NMR spectra and the monomer unit ratios of the copolymer were also calculated by the peak integration values of the spectra.

Additionally, number-average molecular weights (Mn) and molecular weight distributions (polydispersity index, PDI = Mw/Mn) of copolymers were determined by gel permeation chromatography (GPC) using a series of linear Tskgel Super columns (AW3000 and AW5000), with OPTILAB DSP Interferometric Refractometer (Wyatt Technology) as the detector. The eluent was DMF at a flow rate of 1.0 mL min^−1^. Monodispersed polystyrene standards were used to generate the calibration curve. The Mn of the copolymers was in the scope ranging from 63.4 to 79.1 kDa, with the PDI in the range of 1.3 to 1.6.

### 2.3. Preparation of Zwitter-Wettable Polymeric Coatings

The PC plates were cut into rectangular pieces (2.5 × 7.5 cm), and then sonicated in ethanol and water and completely dried in a vacuum oven. The resultant copolymers were dissolved in acetone to get 10 wt.% solutions. After being immersed into the copolymer solution for 60 s, and suspended in the air for 20 s, the copolymer coated PC plates were illuminated by UV lamp for 3 min (300 W, 360 nm, High-pressure mercury lamp, Kangxiao Photoelectric Technology) to get the UV curable coatings. The prepared coatings were washed by acetone, and dried in a vacuum oven at 80 °C, which was nominated as C-30%, C-50%, C-70% C-90%, respectively.

### 2.4. Antifogging and Frost-Resisting Evaluations

The general anti-fogging properties were first tested by a warm breath. The following antifogging test in a high temperature was carried out as follows: A 250 mL beaker with 100 mL DI water was placed on a hot plate and the water was adjusted at 80 °C with thermocouple. When the water temperature is stable, the samples were placed the water vapor with the coated surface facing down. Moreover, the frost-resisting test was executed by storing the samples in a freezer at −20 °C for 2 h, and then the samples were exposed to ambient conditions (~20 °C, 30–40% relative humidity) to observe the frost formation. The surface fogging and frosting behaviors were evaluated by UV-Vis Spectrophotometer (Agilent Cary 60) with a light transmittance in the range of 400–700 nm and the antifogging and frost-resisting phenomenon were recorded by digital camera. To maintain the consistency of experimental conditions, the exposure time was measured within 3 s after the sample was placed between the camera and the test images.

### 2.5. Time-Dependent Surface Wetting Behavior

According to previously reported method [31], contact angle measurements over time were performed for the samples of C-30%, C-50%, C-70%, and C-90%, with a control PC plate a reference. About 10 μL water droplets were added to the sample surfaces, and then the changes of water contact angles as well as the wetting diameters over time were recorded every 4 s during the 80 s incubation period. By analyzing the changes of the contact angle values and the wetting diameters with time, the mechanism of the antifogging behavior for the zwitter-wettable surface can be explained.

### 2.6. Coating Stability

The resultant samples were subjected to three types of tests to evaluate their antifogging stability in different aggressive environments, including UV irradiation, water immersion, and thermal aging treatment. The optimal samples of C-70% were chosen as the representative examples, the changes of the surface antifogging performances after the treatments were evaluated. UV irradiation test was conducted by exposing the coating to a UV lamp with a radiation intensity of 0.8 mW cm^−2^ at λ = ~360 nm for 24 h. For the water immersion test, the samples were socked in DI water (45 °C) for 24 h. Moreover, the coatings were placed in an oven at 100 °C for 24 h to conduct the thermal aging test.

## 3. Results and Discussion

### 3.1. Preparation and Properties of the Zwitter-Wettable Coatings

The zwitter-wettable coating was successfully prepared according to the following procedure. First, the copolymers were synthesized by a thermally triggered free-radical polymerization reaction with the hydrophilic HEA, hydrophobic MMA and 4-BP-acrylate units. Herein, the 4-BP-acrylate groups were served as UV photo-initiator to immobilize the copolymer on the PC substrate surface. The mechanism of the UV triggered surface grafting is that the BP-based groups were excited under UV irradiation to a singlet state and jump to a triplet state which then underwent hydrogen-abstracting reaction from substrates while the resulting BP radicals tended to participate in coupling reaction to covalently bond the copolymers to the substrate surface [43]. By adjusting the mass percentages of the HEA/MMA, the surface zwitter-wettable behaviors can be well controlled. A great number of hydrophilic HEA units in the copolymer backbone could enhance its water adsorption capability, hence improving its antifogging performance. The Mn of the copolymers for P-30%, P-50%, P-70%, P-90% were 79.1 KDa, 71.3 KDa, 69.2 KDa, and 63.4 KDa, respectively, as determined form the GPC test.

**Figure 1:**
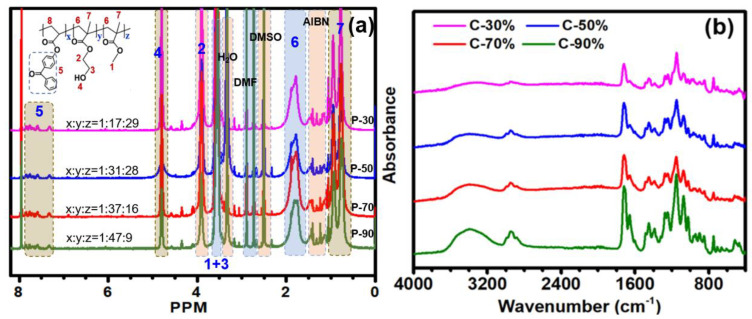
^1^H NMR spectra (**a**) and FT-IR spectra (**b**) of P(HEMA-co-MMA) copolymers.

The chemical structures of the copolymer were investigated by H-NMR analysis (Figure 1a). The methylene (CO-CH_2_-CH_2_) resonance from polyHEA segment appears at 3.9 ppm. The other methylene (−CH_2_-CH_2_-OH) and the methyl protons from poly(MMA) appeared at around 3.6 ppm. Moreover, multiple proton peaks at around 6.8, 7.6, 7.8 ppm were attributed to the benzophenone groups. Taken together, those 1H NMR results confirmed the successful preparation of the copolymers. Additionally, the molar ratios of HEA/MMA/4-BP acrylate in copolymers were also roughly estimated by the peak intergration of the ^1^H NMR spectra which were consistent with the initial feed ratio by conversion to mass fractions as mentioned before. After being treated under UV light irradiation and sufficient cleaning to eliminate the unbonded copolymers, the resulting coatings (C-30%, C-50%, C-70%, and C-90%) were evaluated by ATR-FTIR spectroscopy to check the chemical composition of the surfaces (Figure 1b). The broad band at 3540 cm^−1^ stemming from the −OH stretching showed obvious enhancement due to increased HEA contents in the copolymers. The C = O stretching bands in HEA, MMA, and 4-BP acrylate were also observed at ~1730cm^−1^, indicating the presence of the copolymers on the substrate surfaces.

### 3.2. Antifogging Performances

**Figure 2:**
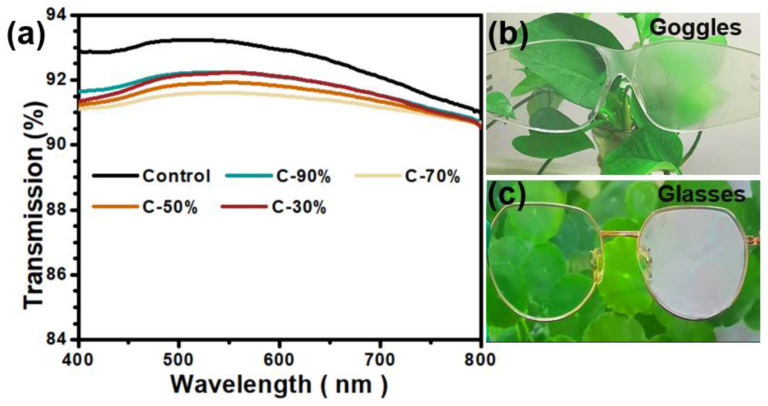
(**a**) Light transmission at the normal incident angle for various samples under ambient conditions. Antifogging performance of treated lens surfaces after a simple breath fogging test on both the PC-based goggles (**b**,**c**) eyeglasses with one lens coated and the other uncoated.

Before antifogging test, the light transmission values of the coatings (C-30%, C-50%, C-70%, and C-90%) were evaluated with the control PC plate as the reference, as displayed in Figure 2a. All the coatings demonstrated high light transmission values (more than 91%), which were comparable to that of the bare PC plate (~92%). The results demonstrated that the introduced coatings on the surface possessed good light transparency, the coating material itself does not cause any significant light absorption. However, just through a simple breath fogging experiment, a clear difference in the antifogging performance of the sample surface can be found. Compared to the blurred picture observed by the control lens, the surfaces treated by antifogging coatings for both the goggles and glasses showed clear views behind the lens (Figure 2b).

**Figure 3:**
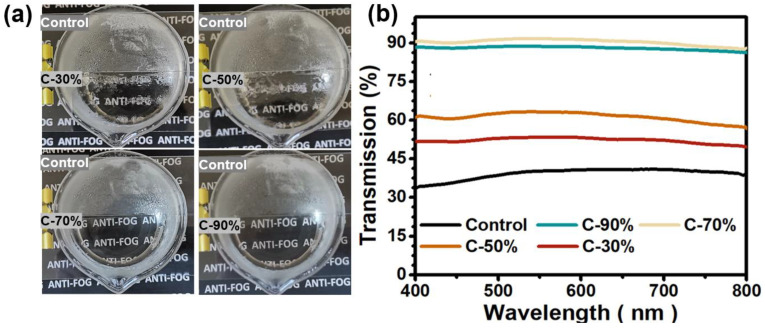
(**a**) Photo images of the samples C-30%, C-50%, C-70%, C-90%, with the control PC plate as a reference, after being exposed to moist water vapor for 60 s (5 cm above an 80 °C water bath). (**b**) The related light transmission over the range of 400–800 nm at the normal incident angle for resultant coatings with the control PC plate as reference.

As one of the most prevalent natural phenomena, fog droplets can form on a variety of surfaces under the right conditions, which can trigger severe light reflection and refraction and reduce the light transmission of transparent substrates. Antifogging surfaces are often considered to be one of the most promising strategies for mitigating fog formation, but their performances can be significantly affected by different fogging environments. In this section, antifogging tests in either hot (80 °C) fog conditions were first conducted, and the related antifogging performances were qualitatively and quantitatively recorded. As for the antifogging test, all the coated PC films were placed above 5 cm over the hot water (80 °C) for 60 s with the coated side face down, the fogging images were recorded with a digital camera and the light transmission were examined by UV-vis spectrophotometer immediately (Figure 3a,b). For the control PC plates, a heavy fog layer rapidly appeared on the downside surface upon exposure to water vapor. Prolonging the exposure time to 60 s made these situation even worse, larger fog droplets stemming from the vapor condensation gradually appeared on the film surface, finally leading to a completely non-transparent film. The fog layer formed caused the background image blurred. When the exposure time was extended to 60 s, dense droplets of fog gradually appeared on the film surface, finally resulting in a completely opaque film. For comparison, the coated samples exhibited quite different antifogging behaviors. The C-30% and C-50% samples showed good anti-fogging performance in the initial period of 5–6 s. As the incubation time was prolonged, a tendency of fog droplet formation and becoming larger began to appear on the surface. When prolonging the incubation time to 60 s, non-continuous hydration layers were formed on both two C-30% and C-50% surfaces. In contrast, both the C-70% and C-90% displayed remarkably enhanced antifogging performances in hot and humid environments.

The antifogging properties of the sample surfaces were also evaluated quantitatively, by immediately collecting the light transmission over the range of 400–700 nm. The overall transmittance results are generally consistent with the anti-fog images, with all samples exhibiting very different light transmissions. Among these, the control PC plates exhibited relatively low transmittance values (~34%) in the 400–800 range, compared to an initial value of ~92% prior to the fogging test. For the coated samples, the antifogging performances of the samples were closely related to the contents of hydrophilic monomers in the copolymers, with a progressive increase in light transmission from 52% to 61% for C-30% and C-50% accompanied by an increase in HEA content. Previous reports have confirmed that the presence of hydrophilic components in the coating can effectively absorb the fog droplets formed on the surface [32]. More hydrophilic components in the coating will result in a stronger water absorption capability, which also enhances the surface antifogging performance. When the HEA mass percentage was increased to 70%, the C-70% coating achieved ~90% light transmission even after fogging test. However, by continually increasing the HEA mass content to 90%, the light transmission of the C-90% showed a slight decrease in light transmission. This phenomenon may be due to the excessive content of hydrophilic component in the coating, which absorbs excessive water vapor and causes excessive swelling of the coating, finally resulting in a reduction of its surface light transmission. Therefore, C-70% proved to be the optimal sample to get the superior antifogging effect against hot moist air.

### 3.3. Frost-Resisting Performances

**Figure 4:**
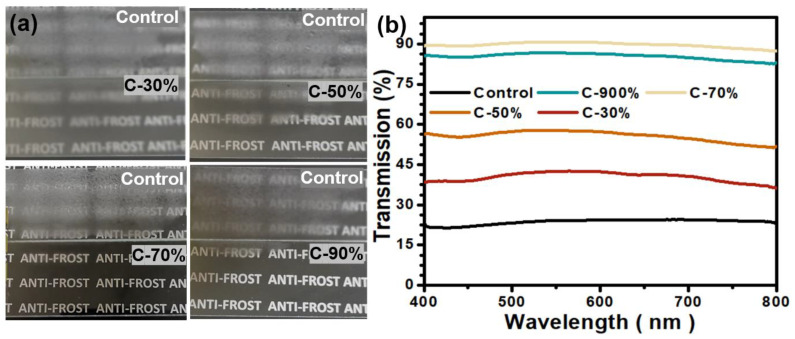
(**a**) Photo images of the samples C-30%, C-50%, C-70%, C-90%, with the control PC plate as a reference, the samples were first stored in a refrigerator (−20 °C) for 2 h, and the photo images were collected after being exposed to ambient conditions (20 °C, 30–35% relative humidity). (**b**) The related light transmission over the range of 400–800 nm at the normal incident angle for resultant coatings with the control PC plate as reference.

Besides the hot temperature antifogging performance, the frost-resisting property is also critical for a functional surface to get more broad antifogging applications. When water vapor in a humid environment comes into contact with a cold surface, the water vapor at the low temperature material interface becomes supersaturated and quickly condenses on the surface to form fog droplets first and subsequently turns into a frost layer due to the extremely low surface temperature. Since this type of frost layer is formed directly from condensed water droplets on the surface, it has a severe reflective and interfering behavior toward light, which greatly reduces the transmittance of visible light. To challenge a harsh fogging situation, the samples were stored in a refrigerator at −20 °C for 2 h and then exposed to the ambient condition (∼20 °C, 35–40% relative humidity) (Figure 4a,b). When the control PC plates were transferred from an extremely cold environment to an ambient environment, a fog layer was formed on the surface immediately, which was then converted into a frost layer due to the cold temperature of the substrate. Both the fog and frost layers formed on the surface have serious impacts on the light transmission of the sample, and only a very blurred image can be observed through the control PC plate. In comparison, the coated samples showed varied surface frost-resisting performances. The C-30% did not display substantial frost-resisting performance and the background image was very unclear. In contrast, the C-50% showed an obviously enhanced frost-resisting behavior, while the C-70% and C-90% samples both displayed significantly enhanced frost-resisting performances, with no fog or frost layer present on the surface during the whole frosting test, although the C-70% sample showed even superior clarity than that of the C-90%. The light transmission confirmed that the frost-resisting performances of the samples were closely related to the hydrophilic HEA contents, both the C-70% and C-90% showed higher light transmissions than 88%. Similar to the antifogging results mentioned above, the coatings with excessive HEA will weaken the frost resistance. Previous studies have confirmed that excessive hydrophilic content in copolymer can lead to excessive water uptake by the coatings, which can produce inhomogeneous water domains during the frost formation at low temperatures, thus remarkably reducing the light transmission to certain extent [30].

### 3.4. Surface Wetting Behaviors

**Figure 5:**
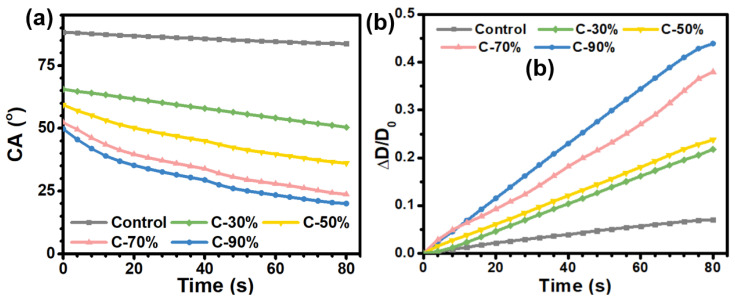
(**a**) Changes of water contact angle on the coating surface within 80 s incubation time. (**b**) Diameter changes of water droplet on coating surface within 80 s, which was expressed as ΔD/Do, where ΔD = D - Do and Do is the initial wetted diameter of the water droplet (time = 0 s) and D is the wetted diameter of the water droplet at certain time. All the data were the average of three times recorded in 4 s interval.

The wetting properties of the sample surfaces have a significant effect on their anti-fogging performances. Among them, the superhydrophilic surface is used to achieve the anti-fogging performance by forming a smooth and uniform hydration thin layer by rapidly spreading fog droplets on the surface, while the superhydrophobic surfaces repel the micro-sized fog droplets from the surface by using the unique super-repellence of the surface. To investigate the anti-fogging performance of the amphiphilic hydrophilic surface, the wettability of the sample surface with time was also investigated. To understand the anti-fogging mechanism of the zwitter-wettable coating surface, the time depended water CA values was monitored, and the changes of the CA values as well as the diameter changes of water droplet on the coating surface were recorded in each 4 s interval within 80 s incubation period (Figure 5).

As shown in Figure 5a, the control PC plate showed relatively high CA values ~88°, and the CA values only showed a slight decrease during the incubation time of 80 s. The result confirmed that the saturated water vapor tends to generate tiny droplets on the hydrophobic surface when it encounters a temperature change. The coated surface also exhibited relatively high initial contact angles, with CA values of ~70°, 65°, 53°, and 49° for C-30%, C-50%, C-70%, and C-90%, respectively, which is quite different from typical superhydrophilic antifogging surfaces with very low water CA (≤5°). These results confirm that an antifogging surface does not need to be superhydrophilic to diffuse condensed droplets into a thin water layer. It was noteworthy that the water CA value of the coated surface varied with time and decreases significantly during the incubation time. The CA of the water droplets on the bare PC plates surface decreased by only 3° during the 80 s incubation time of the water droplet on the surface. In contrast, the CA of the C-30% dropped significantly, from an initial 65° to 50°, with a CA decrease of ~15°. The continuous increase in the content of hydrophilic HEA units resulted in more pronounced decreases in CA values. More significant decreases in CA values, ~20°, 29°, and 30° were observed on the C-50%, C-70% and C-90% surfaces, respectively. These results indicated that the higher content of HEA units in the copolymer could result in a stronger water absorption capacity, which was closely related to the antifogging properties of the surface.

In addition, the variation of water droplet diameter over time on the coated surface was also evaluated (Figure 5b). The coated surfaces with more HEA content exhibited a more pronounced increase in wetting diameter compared to the bare PC surface. Among them, the C-30% and C-50% samples showed ~22% and 24% increase in wetting diameter. Further increase in HEA content led to a continuous increase in wetting diameter, with ~37% and ~43% increase found on the C-70% and C-90% surfaces. This phenomenon further confirmed the fact that the hydrophilic HEA segment in the coating facilitates the adsorption of water, which leads to an expansion of the water wetting area. Together with the anti-fogging results, we can get the conclusion that this zwitterionic wettable coatings with a suitable hydrophilic-hydrophobic balance possess a suitable hydrophilic-hydrophobic balance and thus have excellent antifogging properties.

### 3.5. Surface Stability

**Figure 6:**
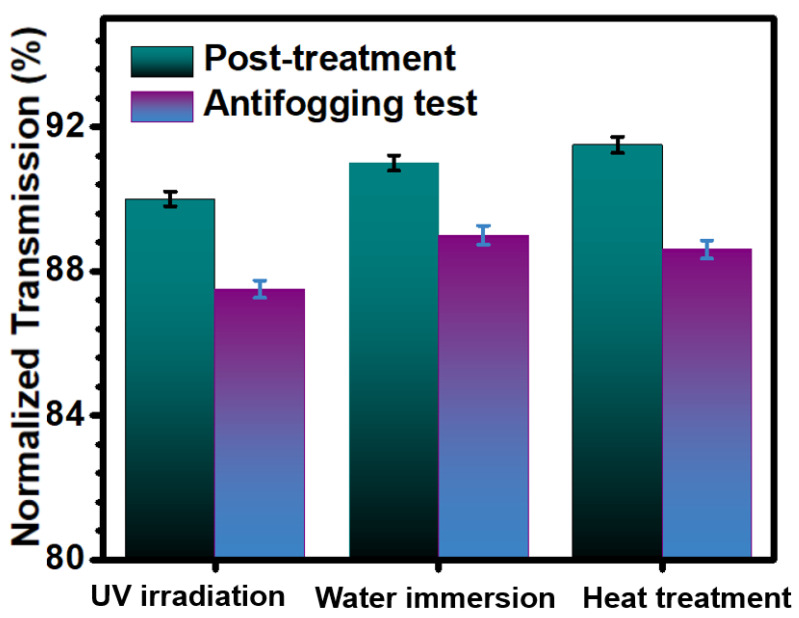
Normalized light transmission of the C-70% under different treatments and its antifogging performances, by exposing the samples to moist water vapor for 60 s (5 cm above the 80 °C water bath).

The surface properties of the coating may be affected when exposed to some harsh environments (e.g., UV irradiation, water immersion, heat treatment), which in turn can lead to a weakening or even loss of surface antifogging functions and seriously affect the lifetime of the functional surface. To verify whether the resulting samples were affected by the above treatments, the C-70% samples were subjected to UV irradiation, water immersion, and heat treatment, respectively, and their surface antifogging properties were quantitatively studied, according to the above-mentioned antifogging test (Figure 6). After 24 h UV irradiation (0.8 mW cm^−2^, 360 nm), the C-70% maintained more than 90% light transmission. The following fogging test confirmed that the antifogging performance of the UV-irradiated samples was slightly reduced compared to its initial antifogging behavior (~90%) of the samples without UV irradiation, but still maintained a high light transmission rate of more than 87%. For those samples immersed in water bath for 24 h, and the surface still maintained relatively high light transmittance (~89%), and also possessed with highly efficient antifogging performance. Moreover, the samples also exhibited excellent thermal stability after being heated in an oven at 100 °C for 24 h, with ~88% light transmission after fogging test. Together, the above results demonstrated that the prepared samples have excellent coating stability and can maintain significant antifogging properties even after being treated by UV irradiation, water immersion, and heating treatment.

## 4. Conclusions

We have successfully developed a zwitter-wettable antifogging and frost-resisting coatings, through a UV-curable copolymer consisting of HEA, MMA, and 4-BP acrylate. Owing to the introduced BP groups in the copolymer chains, the copolymers can be easily bonded on the PC substrate surface upon a convenient UV light irradiation. Because of the suitable hydrophilic-hydrophobic balance, the C-70% was considered as the optimized sample, exhibiting both remarkably high antifogging and frost-resisting performances, with more than 85% light transmission. The antifogging mechanism beneath the coating as revealed by its time-dependent wetting behavior mainly relied on its surface water-adsorbing capability to imbibe condensed water vapor on the surface outmost layer, hence avoiding the surface fog formation. In addition, the stability of the coating was studied by exposing the coating to UV radiation, water immersion and heat treatment, and the results showed that the treated coatings still maintained good antifogging properties. Benefiting from the merits of simplicity, low-cost preparation, and high efficiency of the coating preparation, we believe that this UV-curable acrylate antifogging coating may find a wide range of applications in various display devices, ranging from goggles to medical detection devices.

## Data Availability

The data presented in this study are available upon request from the corresponding author.
